# Opening New Worlds of Meaning—A Scoping Review of Figurative Language in Autism Spectrum Disorder

**DOI:** 10.3390/bs15111556

**Published:** 2025-11-14

**Authors:** Bjørn Skogli-Christensen, Kristine Tyldum Lefstad, Marie Florence Moufack, Sobh Chahboun

**Affiliations:** 1 Department of Education and Lifelong Learning, Faculty of Social and Educational Sciences, Norwegian University of Science and Technology, Campus Dragvoll, Edvard Bulls veg 1, 7491 Trondheim, Norway; bjornwer@hotmail.com; 2 Department of Pedagogy-Section: Special Needs Education, Queen Maud University College, Thrond Nergaards veg 7, 7044 Trondheim, Norway; krity@trondelagfylke.no; 3 Department of Health and Education, Trøndelag Høyere Yrkesfagskole/THYF, Higher Vocational Education, Wessels veg 75, 7502 Stjørdal, Norway; 4 Department of Social Work, Faculty of Social and Educational Sciences, Norwegian University of Science and Technology, Campus Helgasetr Øya, Vangslunds Gate 2, 7030 Trondheim, Norway; marie.f.moufack@ntnu.no

**Keywords:** autism spectrum disorder, figurative language, metaphor, idiom, irony, sarcasm, pragmatics, intervention, education, scoping review

## Abstract

Figurative language (metaphor, idiom, irony/sarcasm) is central to pragmatic communication but is frequently challenging for children and adolescents with autism spectrum disorder (ASD). A scoping review was conducted to map pedagogical and clinical interventions that target figurative-language skills in school-age learners with ASD and to summarize reported outcomes. Following a PCC (Population–Concept–Context) framework and PRISMA-ScR reporting, systematic searches were performed in ERIC and Google Scholar (2010–2025). Eligibility required an ASD sample (ages 5–18), an intervention explicitly addressing figurative-language comprehension, and empirical outcome data from educational or related practice settings. Seven studies met inclusion criteria: five targeting metaphors, one targeting idioms, and one targeting sarcasm/irony. Interventions were predominantly delivered one-to-one or in small groups and emphasized structured, explicit instruction with visual scaffolds and stepwise prompting. Across studies, participants demonstrated clear gains on trained items. Generalization beyond trained material was most often observed for metaphor and sarcasm interventions, particularly when instruction highlighted underlying semantic relations or cue-based pragmatic signals; by contrast, the idiom program yielded item-specific learning with minimal near-term transfer. Limited follow-up data suggested short-term maintenance where assessed. Reported variability across individuals was substantial, underscoring the influence of underlying structural-language skills and social-pragmatic demands. Overall, the evidence indicates that figurative-language skills in ASD are amenable to targeted intervention; effective programs tend to combine explicit teaching, visual supports, multiple exemplars, and planned generalization opportunities. Given small samples and methodological heterogeneity, further classroom-based trials with longer follow-up and detailed learner profiles are needed. The findings support integrating figurative-language goals within individualized education and speech-language therapy plans, while aligning instructional complexity with each learner’s linguistic and pragmatic profile.

## 1. Introduction

Figurative language refers to expressions whose intended meaning extends beyond their literal interpretation. This category includes metaphors (e.g., “X is a rocket” to mean “X is very fast”), idioms, similes, irony, sarcasm, metonymy, and other nonliteral usages. Understanding meaning beyond the literal, as in metaphors, is an important part of children’s cognitive, linguistic, and social development. Figurative competence expands their horizon of understanding and fosters flexible ways of reasoning. For example, grasping the metaphor “time is money” enables a child not only to comprehend an abstract concept such as time management, but also to engage in culturally shared ways of reasoning and communicating about everyday experiences. Mastery of figurative language is therefore a vital component of pragmatic communication and overall language, and moreover cognitive development.

Children and adolescents with Autism Spectrum Disorder (ASD) often experience pronounced difficulties in figurative and pragmatic language (Kalandadze et al., 2018). ASD is a neurodevelopmental condition characterized by persistent deficits in social communication and interaction, alongside restricted and repetitive behaviors (American Psychiatric Association, 2013). The social communication impairments in ASD commonly include challenges in understanding implicit and nonliteral aspects of language. Clinically, many autistic individuals are described as interpreting language in an overly literal manner, struggling with idioms, metaphors, sarcasm, and humor that typical peers grasp intuitively.

In typical development, children gradually learn to interpret nonliteral meanings during middle childhood and adolescence, as their linguistic, cognitive, and social skills mature (e.g., grasping simple metaphors by age 6–7 and more complex forms such as irony during later childhood). By adulthood, figurative expressions are ubiquitous: figurative language is used in virtually all facets of daily conversation, in literature, and in educational materials. For instance, analyses of school textbooks reveal a high density of metaphors and idioms, and understanding these expressions correlates strongly with reading comprehension. Figurative competence also plays a central role in social development. Using and comprehending figurative speech—such as jokes, idiomatic banter, and sarcasm—helps children bond with peers and participate in group interactions. Conversely, a child who interprets language only literally may miss subtle humor or implied meanings, potentially leading to misunderstandings or social exclusion.

Historically, the pervasive figurative language difficulties in ASD led to the common practice of avoiding nonliteral expressions when communicating with this group. For instance, teachers and parents may deliberately use highly concrete language and strictly literal phrasing in order to avoid confusion. While well-intentioned, this avoidance may inadvertently deprive autistic children of opportunities to learn figurative language. Recent perspectives in the field emphasize that figurative language should not be eliminated from the environment of autistic learners, but rather explicitly taught. As Kalandadze (2019) argues, “instead of avoiding using figurative language with these individuals, teachers, parents, clinicians, speech and language therapists should teach comprehension strategies.” This represents a paradigm shift: recognizing that figurative language competence can be improved in ASD through appropriate supports. It also aligns with a broader move toward inclusion, ensuring that neurodivergent individuals can access the richness of everyday language rather than being restricted to overly literal communication.

Figurative-language instruction can serve multiple educational and therapeutic goals, including improving understanding of classroom discourse, enhancing reading comprehension, fostering better understanding of peers, and strengthening everyday social competence.

### 1.1. The Current Study

In light of the above considerations, there is a clear need for systematic documentation of whether interventions targeting figurative language in autism are effective, as well as for the development of approaches that adequately address the needs of children and adolescents with ASD. The present paper undertakes a scoping review of educational and therapeutic interventions designed to support figurative language development in this population.

Whereas prior meta-analyses have quantified the extent of figurative language impairments in ASD (e.g., Kalandadze et al., 2018, who found robust deficits in metaphor and idiom comprehension compared to typically developing peers), considerably less attention has been devoted to identifying and evaluating interventions that may remediate these difficulties.

The guiding research question for this review is therefore: Which educational or therapeutic interventions have been empirically documented as effective in improving the comprehension of figurative language (e.g., metaphors, idioms, sarcasm) among school-age children and adolescents with ASD?

By mapping the available interventions, their strategies, and reported outcomes, this review seeks to highlight practical approaches that practitioners can implement in supporting figurative language learning. Particular emphasis is placed on the ways in which these interventions draw on pragmatic context, social understanding, and language strengths, or alternatively compensate for areas of weakness. Ultimately, the aim is to synthesize insights from the existing literature to inform both future research by identifying knowledge gaps, and to inform applied practice, with the overarching goal of enabling children and adolescents with ASD to develop a richer and more flexible command of language, thereby enhancing their participation in both social and academic contexts.

### 1.2. Theoretical Explanations of Figurative Language Challenges in ASD

Numerous theories have sought to explain why individuals with ASD tend toward literal interpretation and pragmatic difficulties. A useful distinction is that between linguistic pragmatics-inferences derived from language conventions or structural cues, and social pragmatics, inferences that depend on contextual information and the ability to infer speakers’ intentions.

Andrés-Roqueta and Katsos (2017, 2020) argue that autistic children may perform relatively well on linguistic pragmatic tasks, such as resolving ambiguities or understanding metaphors based on linguistic analysis and reasoning, while struggling disproportionately with social pragmatic tasks that require perspective-taking and contextual integration. Empirical studies support this distinction (e.g., Whyte et al., 2014; Norbury, 2004, 2005). Some autistic individuals show near-typical performance on pragmatic inferences such as scalar implicatures (e.g., interpreting some to mean “not all”), novel metaphor or indirect requests, yet exhibit marked deficits in irony or sarcasm comprehension, which hinge on recognizing the speaker’s attitude and the broader communicative context. Petit et al. (2025) similarly report that “some (but not all) pragmatic inferences are difficult for autistic children”, highlighting that those involving social cues, such as irony, are especially problematic. This suggests that figurative language forms requiring mentalizing about others’ beliefs or intention, such as sarcasm or irony, are often the most impaired in ASD, linking figurative challenges to core social-cognitive deficits.

Though the extent to which sarcasm/irony comprehension requires mentalizing remains contested, cue-based, shallow pragmatic processing can sometimes suffice when prosody, facial/gestural signals, or contextual incongruity are salient (Deliens et al., 2018; Chevallier et al., 2011; Kissine, 2012; Kissine et al., 2015). However, when successful uptake depends on a speaker’s specific attitudes or on shared background, deeper intersubjective processing is typically necessary, and jokes often “fall flat” in the absence of such common ground (Kissine, 2012; Kissine et al., 2015; Deliens et al., 2018).

In contrast, figurative forms that rely more heavily on linguistic or structural knowledge may be less affected, particularly among autistic individuals with strong structural language abilities (e.g., vocabulary and syntax). Indeed, an increasing number of studies report successful metaphor comprehension in autistic individuals with intact language skills, underscoring the heterogeneity of figurative competence within this population (Kalandadze et al., 2018). In contrast, children with structural language difficulties often independent of diagnoses show difficulties with interpretation of figurative language.

One influential explanatory framework is Theory of Mind (ToM), which concerns the ability to attribute thoughts, beliefs, and motivations to others. Figurative language frequently requires such perspective-taking. For instance, irony and sarcasm demand an appreciation of the speaker’s intention, which is often opposite to the literal meaning. F. G. E. Happé (1993) demonstrated that individuals with ASD often struggle with these higher-order ToM demands, which may explain their difficulties in interpreting irony. Later research, however, suggests that core linguistic abilities also have strong explanatory power, indicating that figurative language competence in ASD arises from the interplay of both linguistic and social-cognitive factors (Norbury, 2005; Kalandadze et al., 2018).

These perspectives align with findings that pragmatic language competence depends on underlying structural language skills, while at the same time driving broader social communicative behavior. As Volden et al. (2009) and Reindal et al. (2023) argue, strong structural language abilities (e.g., vocabulary and syntax) are necessary prerequisites for pragmatic competence, which in turn provides the foundation for successful social interaction. Figurative language difficulties in ASD therefore cannot be viewed in isolation, but rather as reflecting the dynamic interaction between linguistic resources, pragmatic reasoning, and social cognition. This is in line with studies that supports that children with autism without structural language difficulties can perform as good as TD children.

Moreover, this highlights that figurative language difficulties in ASD are not uniform. Instead, they reflect a complex interaction between structural language skills, pragmatic reasoning, and social cognition. This heterogeneity underscores the importance of tailored pedagogical approaches that account for both linguistic strengths and social-cognitive challenges when designing interventions for figurative language.

### 1.3. Linguistic Versus Social Pragmatics

Pragmatic language abilities in individuals with autism spectrum disorder (ASD) can be conceptualized as comprising two interrelated yet distinct dimensions: linguistic pragmatics and social pragmatics (Andrés-Roqueta & Katsos, 2017, 2020). Linguistic pragmatics refers to the capacity to draw pragmatic inferences based on linguistic form and convention. This includes, for example, understanding implied meanings by relying on familiar vocabulary, grammatical cues, or established linguistic rules. In contrast, social pragmatics involves interpreting a speaker’s intended meaning by integrating contextual information and engaging in perspective-taking, often described in terms of Theory of Mind (ToM).

Research consistently indicates that many individuals with ASD demonstrate relative strengths in linguistic pragmatic reasoning while exhibiting marked difficulties in social pragmatic inference (Andrés-Roqueta & Katsos, 2017, 2020; Norbury, 2005, 2014). In other words, when figurative language comprehension can be anchored in explicit linguistic signals or rule-based patterns, autistic children may successfully compensate by drawing on their structural language skills (Petit et al., 2025). For instance, if figurative expressions are introduced as linguistic puzzles—such as explicitly teaching the definitions of idioms or metaphors—many children with ASD are able to derive nonliteral meanings through logical analysis or memorization. Several studies (e.g., Whyte et al., 2013; Lundblom & Woods, 2012; Mashal & Kasirer, 2011) have documented that some autistic children can master metaphors and idioms in structured, instructional settings, likely by applying these linguistic reasoning strategies.

By contrast, social pragmatic aspects of communication tend to remain more persistently challenging for most individuals with ASD. Comprehending humor, idioms, irony, or indirect hints typically requires the ability to appreciate the speaker’s perspective, attend to emotional cues, and integrate broader contextual information—capacities that are frequently diminished in autism (Andrés-Roqueta & Katsos, 2017, 2020). For example, perceiving the ironic intent behind a statement such as “Well, this is a beautiful day!” uttered during a rainstorm involves evaluating the speaker’s knowledge of the situation and likely intentions, and contrasting this with the literal meaning of the utterance (Vicente & Falkum, 2021). These cognitive operations draw heavily on Theory of Mind and contextual sensitivity—areas in which autistic individuals often experience difficulties.

It is well documented that many children on the autism spectrum misunderstand or fail to notice figurative language forms that rely on social context, such as sarcasm, deception, or certain kinds of humor (F. G. E. Happé, 1993; Kaland et al., 2002; Saban-Bezalel et al., 2019). Historically, such social pragmatic deficits were regarded as relatively resistant to change (F. G. E. Happé, 1993), leading to the assumption that pragmatic impairments in ASD represented stable, trait-like characteristics.

The distinction between linguistic and social pragmatics has important implications for intervention. Effective figurative language instruction for autistic learners may need to address both dimensions: leveraging children’s relative strengths in linguistically oriented reasoning (e.g., teaching the meanings of expressions in a rule-based, explicit manner) while simultaneously fostering their ability to interpret others’ intentions and contextual cues. Supporting both dimensions is essential to enable children to generalize their understanding beyond taught examples and apply figurative language skills in authentic communicative contexts.

While the distinction proposed by Andrés-Roqueta and Katsos between linguistic and social pragmatics offers a valuable heuristic for characterizing pragmatic challenges in autism, it may not fully capture the variability observed across individuals. As noted by Vulchanova et al. (2015), even autistic individuals with average or above-average structural language may show persistent difficulties with metaphors and other forms of nonliteral comprehension that do not explicitly rely on perspective-taking. This indicates that pragmatic difficulties extend beyond social cognition alone and may reflect broader processing-level or cognitive-style differences. Vicente and Falkum (2021) argue that a tendency toward rule-governed, literal interpretation may stem from a broader cognitive style characterized by strong preferences for explicit and predictable information, suggesting that literalism can be linked to rule-following behavior. Recent predictive-processing accounts similarly propose that an overreliance on bottom-up input and attenuated contextual prediction can produce a literal-interpretation bias in autism (Vicente et al., 2024). Moreover, as emphasized by Chahboun et al. (2022), pragmatic and linguistic profiles within the autism spectrum are highly heterogeneous, cautioning against any single explanatory model. Integrating individual variability, cognitive-style and predictive-processing perspectives, and the role of communicative context provides a more comprehensive framework—one that also motivates the following discussion of structured versus spontaneous contexts.

### 1.4. Highly Structured vs. Highly Spontaneous Communication Contexts

Another key theoretical consideration concerns the role of the communication setting, specifically the extent to which external structure and support are available to scaffold pragmatic performance in individuals with ASD. A consistent finding in the literature is that children with ASD often perform markedly differently on figurative language tasks depending on whether these are administered in highly structured or highly spontaneous communicative contexts (Petit et al., 2025).

In structured settings—such as one-on-one therapy sessions or classroom lessons explicitly focused on idioms—the interaction is typically organized, predictable, and rich in external supports. Children may receive clear instructions, visual aids, or step-by-step guidance to help them identify nonliteral meanings. Under such conditions, many autistic children demonstrate substantial improvements in figurative language comprehension; they are often able to concentrate, apply taught strategies, and in some cases perform at levels approaching those of typically developing peers on the targeted tasks. By contrast, in spontaneous and unstructured communicative situations—for example, during informal peer conversations at recess or while listening to figurative expressions embedded in storybook reading—these same children may experience considerable difficulties in grasping figurative meanings. Petit et al. (2025) describe this contrast as one between “highly supported” and “highly spontaneous” pragmatic reasoning. The underlying principle is that the more explicit scaffolding and contextual cues an interaction provides, the easier it becomes for a child with ASD to infer pragmatic meaning. Conversely, as interactions become more fluid and unpredictable, children must rely increasingly on their own spontaneous integrative abilities—skills that are often underdeveloped in autism—to derive figurative interpretations. From a pragmatic perspective, this distinction can be illustrated by considering a student who correctly interprets a metaphor when an adult highlights relevant contextual information or provides a multiple-choice prompt, but who fails to recognize the same metaphor during fast-paced, unscripted peer dialog. The degree of structure in communication thus serves as a crucial moderator of pragmatic performance in ASD (Petit et al., 2025). Structured contexts reveal what a child is capable of under optimal supportive conditions, whereas spontaneous contexts reflect the child’s independent, real-time communicative abilities.

These differences have important implications for intervention design. Effective figurative language instruction may benefit from providing strong scaffolding and explicit teaching during initial stages, followed by a gradual fading of supports to promote independent use. Such a staged approach can help bridge the gap between comprehension in instructional settings and the application of figurative language skills in everyday communicative contexts.

Ultimately, sensitivity to the structured–spontaneous distinction highlights that the true test of pragmatic competence lies in the ability to generalize skills to naturalistic, real-world interactions. Achieving this level of generalization typically requires carefully planned, incremental support rather than assuming it will occur automatically once skills have been acquired in structured contexts.

### 1.5. Cognitive and Language-Based Explanations of Figurative Language Difficulties

The difficulties that individuals with autism spectrum disorder (ASD) experience in understanding figurative language are widely attributed to a combination of linguistic and cognitive factors. Rather than stemming from a single underlying cause, these challenges appear to arise from multiple, intersecting mechanisms that together shape pragmatic development. Prominent explanatory accounts highlight the roles of structural language abilities, Theory of Mind (ToM), central coherence, and executive functioning, each contributing distinct perspectives on figurative language processing in ASD. An integrative theoretical framework considers how these elements jointly influence children’s ability to interpret nonliteral meaning.

### 1.6. Structural Language Abilities

One explanatory avenue emphasizes the foundational role of core language skills. Many autistic children present with co-occurring structural language impairments, such as limited vocabulary, delayed grammatical development, or difficulties in producing coherent narratives. These fundamental language weaknesses can significantly constrain the interpretation of figurative expressions. Comprehending nonliteral language typically presupposes a solid grasp of literal word meanings and syntactic structures.

Empirical research supports this view. Norbury (2005) demonstrated that vocabulary knowledge was a strong predictor of metaphor comprehension in children with ASD: those without additional language delays performed comparably to typically developing peers, whereas those with language delays showed markedly poorer performance. Subsequent studies, including a meta-analysis by Kalandadze et al. (2018), have corroborated these findings. Large-sample studies also reveal substantial overlap between structural and pragmatic language domains. Reindal et al. (2023), for instance, reported that individual differences in grammar and vocabulary accounted for roughly one-third of the variance in pragmatic language skills among children undergoing autism assessments. Similarly, Volden et al. (2009) found significant correlations between semantic–syntactic skills and pragmatic performance in high-functioning autistic children.

Notably, however, Volden et al. (2009) also observed that pragmatic ability itself—the capacity to use language appropriately in context—was the strongest predictor of social competence, above and beyond structural language measures. This suggests that while robust language fundamentals provide an essential foundation for figurative language comprehension, they are not sufficient in isolation; successful pragmatic interpretation also depends on cognitive–social factors. Consistent with this, some scholars argue that pragmatic skills in ASD are partly derivative of structural language (Norbury, 2014), yet pragmatics remains a distinct domain because it requires social reasoning that extends beyond grammar and vocabulary (Andrés-Roqueta & Katsos, 2017). In short, structural language proficiency is a necessary but not exclusive ingredient in figurative language comprehension.

### 1.7. Theory of Mind

Beyond linguistic ability, a range of cognitive explanations have been proposed. One prominent account involves deficits in Theory of Mind—the capacity to infer others’ beliefs, intentions, and mental states. Figurative language frequently relies on implied meanings that must be inferred rather than stated explicitly. Research has long documented associations between ToM difficulties and pragmatic deficits in ASD. F. G. E. Happé (1995) found that autistic children who performed poorly on ToM tasks also struggled with metaphor comprehension, suggesting that difficulties in mentalizing may underlie certain figurative language challenges. Similarly, studies of advanced ToM have shown that verbally fluent autistic individuals may fail to grasp irony or implied meanings that are readily understood by neurotypical peers (Kaland et al., 2002).

### 1.8. Central Coherence

A second cognitive account is the weak central coherence theory (Frith, 1989; F. Happé & Frith, 2006), which posits that individuals with ASD often exhibit a detail-focused cognitive style, processing information in a piecemeal rather than holistic manner. In language processing, this tendency can manifest as excessive focus on the literal meanings of individual words or surface structure, at the expense of integrating contextual cues to derive nonliteral meaning. Vulchanova et al. (2012) found that autistic individuals relied less on semantic and narrative context when interpreting utterances, compared to neurotypical controls. For example, an idiomatic expression such as “kick the bucket” might be interpreted literally—by focusing on “kick” and “bucket”—without recognizing its culturally shared figurative meaning (“to die”). This theory provides a plausible explanation for why many figurative expressions pose difficulties: their interpretation depends heavily on integrating broader contextual information, a process that may not occur spontaneously in ASD (F. Happé, 1999; Norbury, 2005). Educationally, this insight suggests the utility of explicitly teaching how context shapes meaning, for example, through whole–part strategies that highlight the overall discourse before focusing on specific figurative phrases.

### 1.9. Executive Functioning

Executive functions—particularly cognitive flexibility and inhibitory control—represent another relevant cognitive domain. Figurative language comprehension often requires suppressing the initial literal interpretation of an utterance and shifting to an alternative figurative one. Deficits in executive functioning, common in ASD, can manifest as cognitive rigidity and difficulty shifting strategies. Empirical observations indicate that autistic individuals may perseverate on literal meanings even when contextual cues signal the need for reinterpretation. Furthermore, high cognitive load can exacerbate these difficulties: according to cognitive load theory (Sweller, 1994), complex figurative expressions may exceed the processing resources of children with executive weaknesses, preventing successful reinterpretation. Instructional approaches that reduce cognitive load—by breaking figurative expressions into manageable components, using visual supports, and allowing additional processing time—may therefore facilitate better outcomes for these learners.

### 1.10. Integrative Perspectives and Individual Differences

Crucially, these explanatory frameworks are not mutually exclusive but likely interact to shape each individual’s pragmatic profile. Pragmatic competence can be understood as emerging at the intersection of linguistic knowledge and social-cognitive processing (Volden et al., 2009). For example, one child’s figurative language difficulties may primarily reflect limited vocabulary and syntax, whereas another child with strong structural language may still interpret utterances literally due to weaknesses in ToM or central coherence.

Any theoretical discussion must also acknowledge the heterogeneity of the autism spectrum. Children and adolescents with ASD exhibit substantial variability in both structural language and pragmatic abilities (Reindal et al., 2023). Some have age-appropriate grammar and vocabulary, while others experience significant language delays; likewise, pragmatic challenges range from subtle to profound. Reindal et al. (2023) reported that approximately 84% of a large clinical sample exhibited some form of language impairment, but the severity and combination of deficits varied widely. This heterogeneity underscores the importance of individualized assessment and intervention: a child’s difficulty with figurative language may stem from different underlying causes—limited vocabulary, lack of relevant experience, or social-cognitive difficulties—depending on the profile.

### 1.11. Clinical and Educational Implications

Studies of figurative language in ASD reveal that pragmatic competence is often uneven, both relative to neurotypical peers and across different figurative forms (Vulchanova et al., 2015), resulting in what has been described as a “bumpy profile.” From a clinical and educational perspective, acknowledging this variability leads to the principle of individualized intervention planning. Best-practice guidelines emphasize that assessments should encompass both core autism symptoms and detailed language profiles (Chahboun et al., 2022), using standardized measures of structural language (e.g., vocabulary, grammar) and pragmatic abilities (e.g., parent-report instruments such as the Children’s Communication Checklist-2; Bishop, 2003, and naturalistic observations) (Reindal et al., 2023).

In summary, understanding figurative language difficulties in ASD requires a multifactorial framework that integrates linguistic, cognitive, and social dimensions. Structural language proficiency provides an essential foundation, while ToM, central coherence, and executive functions modulate the ability to move beyond literal interpretation. Importantly, the diversity of linguistic–pragmatic profiles among autistic individuals necessitates tailored interventions that build on each child’s strengths and address specific areas of need. With appropriate support, many children and adolescents with ASD can expand their figurative language competence, enhancing both academic learning and social participation.

## 2. Method

### 2.1. Design and Reporting Framework

A scoping review methodology was employed to systematically identify, map, and synthesize existing research on pedagogical interventions designed to support the development of figurative language skills in children and adolescents with autism spectrum disorder (ASD). Scoping reviews are particularly appropriate for areas where the evidence base is diverse, emerging, or methodologically heterogeneous, and where the aim is to provide an overview of available knowledge and knowledge gaps rather than to statistically evaluate intervention effects through meta-analysis. This approach was chosen because studies on figurative language interventions for individuals with ASD are distributed across multiple disciplines, including education, linguistics, psychology, and clinical sciences, and encompass a wide range of methodological designs, participant populations, and outcome measures.

The review followed the revised framework proposed by Levac et al. (2010). In line with this framework, the process involved several interrelated stages. First, the core research question was carefully defined, focusing on identifying which educational or therapeutic interventions have been documented as effective for improving the comprehension of figurative language in school-age children and youth with ASD. Second, relevant studies were identified through systematic searches of selected databases and supplementary sources. Third, records were screened and selected for inclusion on the basis of predefined eligibility criteria. Fourth, data from each included study were charted using a structured extraction framework to capture essential information on study characteristics, intervention components, and outcomes. Finally, the data were analyzed and synthesized using both descriptive and thematic approaches to provide a comprehensive overview of the field.

The review adhered closely to the PRISMA-ScR guidelines (Preferred Reporting Items for Systematic Reviews and Meta-Analyses extension for Scoping Reviews), which offer a standardized structure for reporting scoping reviews to ensure methodological transparency and replicability. All stages of the identification, screening, eligibility assessment, and inclusion process were documented, and the study selection pathway is presented in a PRISMA-ScR flow diagram (see Appendix A).

To strengthen the rigor of the search process, pilot searches were conducted to refine search terms, Boolean operators, and database-specific strategies prior to the final searches. The inclusion criteria were then systematically applied to all retrieved records. Data extraction followed a standardized template developed for this review, enabling consistent collection of key variables such as study design, participant characteristics, targeted figurative language domains, intervention strategies, duration and format, outcome measures, and reported effects. The synthesis involved a combination of descriptive mapping to outline the scope and characteristics of existing interventions, and thematic analysis to identify cross-cutting patterns, pedagogical principles, and implications for research and practice.

### 2.2. Information Sources and Search Strategy

Systematic literature searches were conducted using the ERIC and Google Scholar databases. The most recent search was completed on 25 January 2025, and the publication time window was restricted to the period from 2010 to 2025 in order to capture contemporary developments in intervention research. ERIC was selected for its comprehensive coverage of education-focused studies, whereas Google Scholar was included to access relevant interdisciplinary literature spanning psychology, linguistics, special education, and related fields.

The search strategy was developed in accordance with the PCC framework and refined through pilot testing and iterative adjustment to maximize both precision and recall. Boolean operators (AND/OR), truncation, and phrase searching were employed to structure the queries systematically. In ERIC, controlled descriptors (e.g., Autism Spectrum Disorder; Figurative Language; Intervention/Training) were combined with free-text keywords targeting figurative language phenomena (e.g., metaphor; idiom; irony; sarcasm) and intervention concepts (e.g., intervention; training; outcome; effectiveness). Parallel strategies were applied in Google Scholar, using flexible keyword combinations to accommodate the platform’s less structured indexing. These iterative refinements ensured that the final searches achieved broad coverage of the relevant literature while maintaining a high level of specificity.

### 2.3. Eligibility Criteria (PCC Framework)

Eligibility criteria were operationalized using the PCC framework (Population–Concept–Context), which provided a transparent structure for the screening process. The population of interest comprised children and adolescents aged 5–18 years with a formal diagnosis of ASD and sufficient cognitive and linguistic abilities to engage in figurative language tasks. The concept focused on pedagogical or therapeutic interventions explicitly targeting the comprehension of figurative language forms, including metaphors, idioms, irony, and sarcasm. The context encompassed empirical studies conducted in educational or clinical settings that were relevant to language learning and social communication. This framework facilitated a systematic and replicable approach to study selection. To ensure data quality, eligible publications were required to be peer-reviewed journal articles, doctoral theses or intervention reports. Studies were excluded if they (a) did not involve children/young people with ASD, (b) did not assess participants’ language function and IQ, (c) did not report outcomes pertaining to the effect of the intervention, or (d) published in another language than Norwegian or English.

### 2.4. Study Selection

The search identified 393 records (360 from Google Scholar; 33 from ERIC). After removal of 11 duplicates, 382 records were screened (title/abstract), and 32 were retrieved for full-text review. Screening and full-text assessment were undertaken by a single selector. Of the 32 full texts, 5 met the inclusion criteria; 2 additional studies were identified through manual reference-list searches, yielding 7 included studies in total. The full selection pathway (including reasons for exclusion at full text) is shown in the PRISMA-ScR diagram and accompanying table in the thesis Appendix A.

### 2.5. Data Extraction and Synthesis

From each included study, data were charted into an extraction template summarizing: authors and year, sample size and participant characteristics (including functional level), figurative-language targets (e.g., metaphors, idioms, sarcasm), intervention description (approach, strategies, format, duration), outcome measures, and principal findings. Given heterogeneity in designs and outcome metrics, a descriptive narrative synthesis was conducted rather than quantitative pooling. The analysis combined a descriptive overview with thematic analysis (Braun & Clarke, 2006) to identify cross-study patterns in strategies and reported opportunities for practice implementation. The thematization emphasized pragmatic interpretation of authors’ descriptions and grouped findings under (1) linguistic dimensions (metaphor, idiom, sarcasm), (2) theoretical frameworks (behavior-analytic vs. linguistic–cognitive approaches), and (3) generalization/transfer of skills. Elements of a hermeneutic stance were applied in interpreting and relating study findings to illuminate mechanisms and outcomes of the interventions.

### 2.6. Critical Appraisal and Ethics

Consistent with the aims of a scoping review, no formal quality-based exclusion criteria were applied. All studies meeting the predefined inclusion criteria were retained irrespective of methodological limitations, and potential threats to validity were addressed during the synthesis phase. As the review relied exclusively on published data, ethical approval was not required.

### 2.7. Results and Discussion

The scoping review identified seven intervention studies (Persicke et al., 2012; Lee et al., 2019; Mashal & Kasirer, 2011; Whyte et al., 2013; Lynch, 2024; Persicke et al., 2013; Melogno et al., 2017) targeting figurative language in children and adolescents with ASD. Five studies focused on metaphor comprehension, one on idioms, and one on sarcasm/irony. Interventions were delivered in one-on-one or small-group formats, generally emphasizing structured, explicit instruction. Theoretical frameworks varied: most metaphor and sarcasm studies were grounded in behavioral/Relational Frame Theory (RFT) approaches (Persicke et al., 2012, 2013; Lee et al., 2019; Lynch, 2024), whereas a few used cognitive-linguistic strategies (Mashal & Kasirer, 2011; Melogno et al., 2017; Whyte et al., 2013). Across all studies, children showed clear learning on the trained material.

### 2.8. Metaphors

Five studies (Persicke et al., 2012; Lee et al., 2019; Mashal & Kasirer, 2011; Melogno et al., 2017; Lynch, 2024) examined interventions for metaphor understanding. In every case, targeted instruction led to large gains on trained expressions. For example, Persicke et al. (2012) conducted an intensive one-on-one multiple-exemplar training on simple nominal metaphors with three young children (ages 5–6). All children moved from virtually zero correct responses at baseline to near-ceiling accuracy after training. Similarly, Lee et al. (2019) used structured worksheets and storytelling prompts to teach three children (ages 5–8) to identify and explain concrete and abstract metaphors. Two of the three children completed the full program, and both showed steep improvement on the trained metaphors, with accuracy rising to very high levels by the end of intervention. Notably, in these studies improvement on trained items generalized to novel metaphors. Persicke et al. reported that children not only mastered the specific metaphors taught, but also correctly interpreted new, untrained metaphorical expressions in post-testing. Lee et al. likewise found that children could handle novel metaphors after the program.

In a larger group-based study, Mashal and Kasirer (2011) trained 20 children with ASD (age 12–13) to use visual “thinking maps” to analyze metaphors in small groups. After instruction, the ASD group significantly improved on interpreting conventional metaphors compared to baseline. However, this group did not show gains on novel metaphors without explicit practice. In contrast, their comparison group with learning disabilities did generalize to new items. This suggests that the explicit mapping strategy aided the ASD group in understanding taught metaphors, but additional supports (e.g., further guidance or modeling) were needed to generalize to unfamiliar metaphors.

Melogno et al. (2017) reported a single-case intervention with one high-verbal 8-year-old with ASD. The boy was taught a novel strategy for “X is Y” metaphors: he learned to reformulate them as similes (“X is like Y”) and identify shared properties of X and Y. Before training he interpreted metaphors literally (e.g., “a star is not a person”); afterwards he showed dramatic gains. Post-intervention he could generate plausible figurative meanings for new metaphors he had never heard, indicating broad generalization. Remarkably, after training this child also began using more figurative language spontaneously in daily speech—for example, inventing his own metaphors and giving objects metaphorical names, a qualitative shift in his expressive language.

Lynch (2024) addressed metaphors of emotional state (e.g., “It’s a gray day” to mean feeling sad) in three adolescents. A hierarchical, behavior-analytic program first taught emotion vocabulary (e.g., identifying sadness in pictures), then explicitly linked emotions to metaphorical statements. Initially the first training attempts were too difficult, so the procedure was modified to add visual supports and extra scaffolding. Ultimately one participant mastered the task: he achieved high accuracy both on the metaphors explicitly taught and on new emotional metaphors in the same category. In other words, he generalized to novel items (e.g., understanding a metaphor for “happy” after learning a metaphor for “sad”). This effect was maintained—his performance remained high at follow-up. The other two participants did not complete the program, highlighting individual variability; nonetheless, the successful case shows that even abstract metaphor comprehension can be taught given appropriate supports.

Summary (metaphors): All five studies report significant improvements on trained metaphors. In most (Persicke et al., 2012; Lee et al., 2019; Melogno et al., 2017; Lynch, 2024), children also showed transfer to untrained metaphors, demonstrating emergent understanding rather than rote memorization. Two studies (Persicke et al., 2012; Melogno et al., 2017) noted spontaneous metaphor use post-intervention, indicating a qualitative language shift. Lee et al. (2019) explicitly tested maintenance: learned metaphor skills remained stable at a two-month follow-up. In contrast, Mashal and Kasirer (2011) found that without extensive prompting, children with ASD applied the taught strategy mainly to practiced expressions, showing minimal generalization. Overall, these findings suggest targeted metaphor training can yield broad gains in comprehension, especially when instruction highlights semantic relations (e.g., shared features) and provides ample exemplars.

### 2.9. Idioms

Only one study (Whyte et al., 2013) targeted idioms. This group-based intervention was delivered during a two-week summer social skills camp for ten children with ASD (ages 7–12). Nine conventional idioms (e.g., “not a bird, not a fish”) were taught via short stories, direct explanation, interactive worksheets (writing, drawing, coloring), and instructor-guided discussion. At pre-test the children’s comprehension of idioms was extremely low. After the 14-day program, all participants learned the taught idioms: they could explain the correct meanings of each of the nine expressions with high accuracy. A delayed post-test, conducted two weeks later, showed retention of these meanings, indicating the learning was maintained at least over the short term. Crucially, the study also assessed generalization using nine control idioms that were not taught. The children showed no improvement on these untrained idioms (performance remained near floor), and their post-test scores were significantly higher for trained versus untrained items. In other words, instruction produced item-specific gains: the children learned the exact idioms that were practiced, but this knowledge did not transfer to unfamiliar idiomatic phrases. No data on spontaneous idiom use were reported. Thus, Whyte et al. demonstrate that explicit, brief training can expand the idiom repertoire of children with ASD, but the effect is essentially learning by rote, with little immediate generalization beyond the taught set.

### 2.10. Sarcasm

One study (Persicke et al., 2013) addressed sarcasm comprehension, a complex pragmatic skill. In this single-case experimental design, three young boys with ASD (ages 6–7) received behavioral training to recognize sarcastic tone and context. Through video modeling and in vivo role play, they practiced detecting when a speaker meant the opposite of literal meaning, and they rehearsed appropriate responses. After training, all three participants showed marked improvement: they correctly identified sarcastic remarks and chose suitable responses according to the speaker’s true intent. Importantly, learning generalized beyond the therapy context. The boys could detect and interpret novel sarcastic comments (not used in training), and they did so with different people and in different settings. Follow-up testing at three months revealed that the gains were sustained for at least two of the children. In sum, structured explicit instruction enabled these children to grasp the pragmatics of sarcasm, with both generalization to new examples and longer-term maintenance of skills.

### 2.11. Summary of Intervention Strategies and Outcomes

Across the seven studies, effective interventions shared common features. They all used explicit, structured teaching: concepts were taught gradually (often hierarchically), with clear steps and modeling of the reasoning process. Visual supports and semantic strategies were frequently employed. For instance, Persicke et al. (2012, 2013) had children list properties of concepts on a two-column chart to visually link metaphor components; Mashal and Kasirer (2011) used graphic thinking maps; Lee et al. (2019) presented metaphors with pictures and text on slides; Melogno et al. (2017) simplified metaphors into similes with concrete illustrations. Modeling by the instructor was used consistently: therapists demonstrated how to derive a non-literal meaning (e.g., enumerating features to see the shared attribute) before requiring the child to try. Some programs provided multiple exemplars (multitude exemplars of metaphors in different settings and situations with different people) and immediate feedback. For example, Persicke et al. (2012) repeatedly practiced many different metaphor sentences in succession (multiple-exemplar training) and only proceeded when the child succeeded; Lee et al. (2019) used three-step prompts (labeling, describing features, explaining meaning) with echoic and picture cues. In cases where initial attempts failed, researchers added extra scaffolds: Lynch (2024) introduced more visual cues after the first attempt faltered, and Lee et al. (2019) provided additional trials to ensure mastery.

Regardless of the theoretical orientation (RFT behavior analysis vs. cognitive strategy), all approaches yielded learning. Table-based behavior-analytic programs (Persicke et al., 2012, 2013; Lee et al., 2019; Lynch, 2024) effectively taught both metaphors and sarcasm by treating the relations (e.g., coordination of features) as operant skills. Cognitive-linguistic methods (Mashal & Kasirer, 2011; Melogno et al., 2017; Whyte et al., 2013) were also successful when they broke down figurative expressions into comprehensible parts. This diversity indicates multiple viable paths to improve non-literal language. In every study, the primary outcome was consistent: participants improved on the specific figurative forms practiced in the intervention. Generalization of learning varied by context. Metaphor interventions often led to transfer, especially when the training emphasized underlying semantic links. Persicke et al. (2012), Lee et al. (2019), Melogno et al. (2017), and Lynch (2024) all reported that children applied the taught strategy to interpret new metaphors. In two single-case studies (Persicke et al., 2012; Melogno et al., 2017), this emergent understanding was accompanied by spontaneous metaphor production, implying deeper conceptual change. The group metaphor intervention (Mashal & Kasirer, 2011) showed partial transfer: ASD participants improved only on conventional metaphors that resembled the taught items, but not on unrelated novel metaphors. Idioms, however, behaved differently: Whyte et al. (2013) found no generalization. Children memorized the nine idioms taught but could not infer the meanings of nine control idioms. This aligns with theory: idioms are highly idiosyncratic and require lexical learning, so short instruction enlarges known vocabulary without creating inferential rules. Finally, sarcasm training produced excellent generalization. Persicke et al. (2013) observed that after learning to detect tone and context cues, the children could recognize sarcasm in entirely new sentences and from new speakers. This suggests that the pragmatic skill (spotting the speaker’s intent) was internalized to some degree, rather than confined to specific examples.

Maintenance of skills was documented only in a few cases. Lee et al. (2019) re-tested participants after two months and found metaphor comprehension remained high. Whyte et al. (2013) conducted a 2-week follow-up, finding no decay in learned idioms. Persicke et al. (2013) did a 3-month follow-up on sarcasm and saw sustained improvement in at least two children. No study reported relearning declines on the trained material, suggesting that when children with ASD do learn figurative forms, the knowledge is retained at least in the short term.

In sum, the results indicate that targeted pedagogical interventions can effectively boost figurative language skills in ASD. All included studies reported clear gains on the forms taught, and most found evidence of generalization and some maintenance. Explicit instruction, visual scaffolds, and practice across multiple examples emerged as key components. Variability in outcomes (e.g., between-trained and untrained forms, and between individuals) underscores that while ASD children can learn figurative language, the scope of learning depends on both the nature of the expression (metaphor vs. idiom) and the learner’s abilities.

## 3. Discussion

The present findings demonstrate that children and adolescents with ASD are capable of learning figurative language when given appropriate instruction. This challenges the notion that autistic learners are “stuck” in literal interpretation; instead, it suggests that figurative comprehension can be explicitly taught as an operant behavior or cognitive skill. The following section considers these results in light of broader theoretical and clinical framework and highlights their implications for education and therapy.

## 4. Theoretical Interpretation

### 4.1. Figurative Language and Language Abilities

Consistent with prior research, performance on figurative tasks appears strongly tied to general language and cognitive skills. The meta-analysis by Kalandadze et al. (2018) found that group differences in figurative comprehension were minimized when ASD and control children were matched on structural language. Likewise, Reindal et al. (2023) notes that individuals with ASD display profound heterogeneity in language: some are minimally verbal, others are highly verbal with intact phonology, morphology and syntax. In this review, several studies did not report detailed language profiles, but those that did often involved children with adequate vocabulary and conceptual knowledge (e.g., grade-level reading ability in Lee et al., 2019). The success of interventions, especially in metaphor and sarcasm, may reflect that these participants had sufficient semantic resources to benefit. Kalandadze et al. (2018) emphasize that “core language skills” are critical for figurative language, the results align with this. For example, in Lynch (2024), initial failure to learn emotion metaphors was overcome by providing additional support, suggesting that without a strong base in emotional vocabulary the task was too abstract.

### 4.2. Behavioral Versus Cognitive Mechanisms

The included studies span two intervention traditions. Behavioral/RFT approaches conceptualize figurative meaning as a derived relational response: a child learns to treat “X is Y” sentences as an equivalence if X and Y share a feature. Under this view, intensive trial-and-error training with feedback can shape the correct response topography. Indeed, Persicke et al. (2012, 2013), Lee et al. (2019), and Lynch (2024) taught children to list features of each noun and then find the common feature, effectively operationalizing the metaphor as a matching task. These programs treated context and intonation (in sarcasm training) as discriminative stimuli that signal the nonliteral meaning. The success of these methods confirms that relational framing skills can be taught in ASD, supporting RFT accounts of figurative language (as in Persicke et al., 2012).

Despite differing rationales, procedural overlap is substantial. Instructors present worked examples (modeling as an instructional prompt), use graduated prompts, provide repeated practice across exemplars, and deliver immediate feedback. In Persicke et al. (2012), the instructor modeled the feature-mapping steps for “X is Y” metaphors as an antecedent prompt within an ABA/RFT protocol, followed by multiple-exemplar training with contingent feedback. By contrast, within a cognitive-linguistic stance, Melogno et al. (2017) explicitly shaped the shift from simile to metaphor—recasting “X is Y” as “X is like Y” to highlight the shared attribute, then scaffolding the return to predicative “X is Y”—thus easing pragmatic interpretation without relying solely on contingency shaping.

## 5. Implications for Practice

Integrating figurative language into intervention. The clear pedagogical implication is that figurative language should not be excluded from instruction for learners with ASD, as evidence shows that it can be explicitly taught and meaningfully acquired.

Despite historical views that saw this skill as unteachable for ASD, the evidence shows that children can learn nonliteral language with deliberate training. Teachers and clinicians can draw on the strategies identified here: break down metaphors into concrete comparisons, use visual organizers to illustrate relationships, model the inferential steps aloud, and provide repeated practice with diverse examples. For instance, using charts to list attributes of each metaphor element (as in Persicke et al., 2012) or converting metaphors into similes (as in Melogno et al., 2017) are practical techniques. Importantly, interventions should include generalization practice: mix in untrained expressions during teaching or gradually reduce prompts, to encourage learners to apply the rule beyond memorized items. The differential findings on idioms suggest that idiom instruction may be more like teaching vocabulary: children will likely need many examples over time, possibly with visual aid or contextual cues, to internalize idiomatic meanings more broadly.

### 5.1. Accounting for Individual Differences

Given the heterogeneity of ASD, educators or therapist should tailor figurative language goals to each child’s profile. Those with stronger receptive vocabulary and verbal reasoning are likely to benefit more readily from abstract training, while others may need more concrete supports (e.g., pictures, real-life enactments, multisensory cues). Pre-assessment of structural language skills can guide expectations. For children with delayed syntax or limited semantics, it might be necessary to build foundational language first (e.g., teaching key vocabulary, concept knowledge) before or alongside figurative training. The included studies generally involved children without severe language impairment, which suggests these methods may be most feasible in learners at least in the moderate range of functioning. However, even minimally verbal students could potentially engage in simplified versions (e.g., matching pictures to convey “sad” and “rainy” for a simple emotion metaphor). In all cases, patience and systematic progression (as in Lynch, 2024) are crucial.

Furthermore, social-pragmatic readiness can vary: if a child has significant ToM or social-communication deficits, therapists might need to explicitly teach the pragmatic cues for instances of irony or sarcasm (tone of voice, facial expression) before expecting advanced use of ToM skills. The sarcasm study (Persicke et al., 2013) illustrates this: they explicitly trained children to recognize sarcastic prosody and context, then link it to intent. This suggests that for high-level forms of figurative language, underlying pragmatic skills (e.g., perspective-taking) should be included in the training sequence. Ongoing progress monitoring is advised so that instruction can be adapted if a child seems stuck on a particular abstraction level.

### 5.2. Role of Structural Language and General Curriculum

The meta-analytic evidence emphasizes that core language ability underpins figurative competence. Thus, schools should ensure that language interventions for ASD address syntax, vocabulary, and semantics robustly as part of the general plan, improvements there need to be met before expecting figurative understanding. This review suggests that targeting figurative language specifically adds value beyond general language support. For instance, teaching a metaphor strategy (e.g., linking common features) might simultaneously reinforce vocabulary and semantic reasoning, yielding dual benefits. Collaboration between speech-language therapists, special educators, teachers and clinical trainers is important: figurative language training fits naturally within language therapy curricula, but it also has social-communication implications (e.g., understanding humor in peer interactions), so behavioral or social skills programs could integrate these components.

### 5.3. Explicit Instruction Feasibility

All reviewed interventions were intensive and highly structured, often delivered by researchers or therapists outside typical classroom routines. A key question is how feasible such programs are in everyday educational settings. Several points encourage optimism. First, many of the strategies (visual supports, modeling, step-by-step prompts) are already recommended in ASD pedagogy. Teachers can incorporate these into existing language lessons without needing entirely new technology. For example, a reading comprehension activity on a story could explicitly point out any metaphors or idioms and work through their meanings together with students using worksheets and discussion, as was done in Whyte et al. (2013). Similarly, video modeling or group role-plays (as used for sarcasm) can be integrated into social skills groups.

Second, the generalized outcomes in many studies suggest that not all instruction has to be one-on-one. The group metaphor training (Mashal & Kasirer, 2011) and the group idiom camp (Whyte et al., 2013) were group based, indicating that peer-supported settings can work if staff guide the activities carefully. Schools often run social thinking or language arts circles, which could be venues for figurative language modules.

Nevertheless, there are challenges. The interventions reported were relatively short (weeks to a few months) but intensive. Scaling this up might require training more staff in these methods and finding curricular time. One practical approach is to embed figurative language goals into individualized education plans, with measurable objectives. For example, a teacher might track a student’s ability to explain metaphors in regular reading assignments, and provide mini-lessons when idioms appear. Ongoing reinforcement (using learned idioms in conversation, revisiting familiar metaphors) could help maintain gains. The demonstrated maintenance over weeks suggests that once learned, figurative forms need not be practiced daily to persist, but periodic review is advisable.

## 6. Conclusions

In summary, this review finds that figurative language deficits in ASD are amenable to intervention. Both behavioral and cognitive methods can equip autistic learners with strategies for understanding metaphors, idioms, and sarcasm. Consistent features of successful programs were explicit teaching, visualization of abstract concepts, and systematic practice across diverse examples. While idioms may require many exposures to internalize, metaphors and pragmatic forms like sarcasm can often be generalized with conceptual training. These results reinforce the idea that targeted instruction, tailored to each child’s language profile, can close the gap in nonliteral language skills and thereby support richer communication. Educators and clinicians are encouraged to incorporate figurative language goals into their practice, using structured techniques informed by this evidence. With an awareness of individual variability (in language level and learning style) and a commitment to ongoing practice, children with ASD can learn to navigate the figurative speech that is so prevalent in everyday life.

### Gaps in the Existing Literature & Future Directions

The existing evidence, while encouraging, is limited by small samples and lack of control groups in most studies. The generalizability of these findings to broader ASD populations remains to be tested. Moreover, little is known about long-term retention beyond a few months, or about how learning integrates into naturalistic communication. Future research should evaluate classroom-based implementations and examine which components are essential (e.g., is the structured protocol necessary, or would simpler exposure help?).

All seven studies used explicit, structured teaching. No studies showed evidence of pragmatic abilities gained in real life situations. There is a need for research on possibilities for figurative language skills learning in unformal settings.

Importantly, given the heterogeneity of ASD, future studies should report individual differences. For instance, do children with higher vocabulary or nonverbal IQ learn faster? Do some learners become confused when switching from literal to figurative tasks? Monitoring and adjusting for such differences will make interventions more precise.

## Figures and Tables

**Figure A1:**
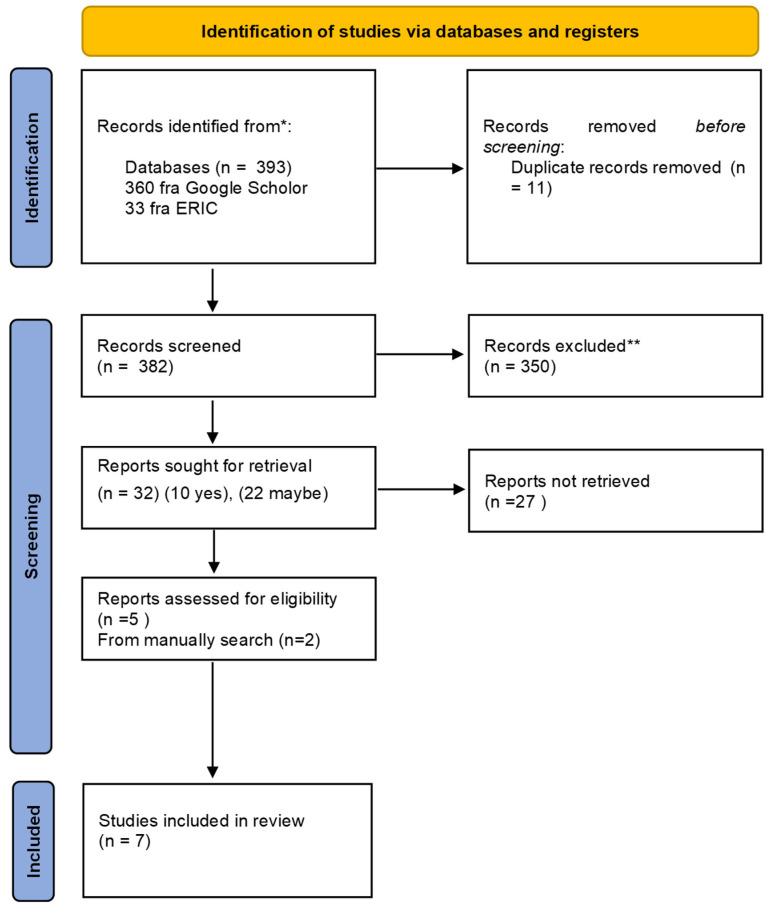
PRISMA-ScR flow diagram illustrating the study identification, screening, eligibility, and inclusion process. Source: Page et al. (2021), BMJ 372:n71. doi: 10.1136/bmj.n71. * Records excluded after title and abstract screening because they did not meet the PCC eligibility criteria (e.g., wrong population, no intervention, no figurative-language outcomes, non-empirical sources). ** Full-text articles excluded with reasons, including: not reporting figurative-language outcomes; inadequate assessment of language/IQ; non-intervention studies; or wrong language (non-English/Norwegian).

## Data Availability

All data supporting the conclusions of this review are contained within the article and Appendix A. Full search strings and the data-charting template are available from the corresponding author upon reasonable request.
